# Engineering Tree Seasonal Cycles of Growth Through Chromatin Modification

**DOI:** 10.3389/fpls.2019.00412

**Published:** 2019-04-05

**Authors:** Daniel Conde, Mariano Perales, Avinash Sreedasyam, Gerald A. Tuskan, Alba Lloret, María L. Badenes, Pablo González-Melendi, Gabino Ríos, Isabel Allona

**Affiliations:** ^1^Centro de Biotecnología y Genómica de Plantas, Instituto de Investigación y Tecnología Agraria y Alimentaria, Universidad Politécnica de Madrid, Madrid, Spain; ^2^HudsonAlpha Institute for Biotechnology, Huntsville, AL, United States; ^3^Oak Ridge National Laboratory, Center for Bioenergy Innovation, Oak Ridge, TN, United States; ^4^Instituto Valenciano de Investigaciones Agrarias, Moncada, Spain; ^5^Departamento de Biotecnología-Biología Vegetal, Escuela Técnica Superior de Ingeniería Agronómica, Alimentaria y de Biosistemas, Universidad Politécnica de Madrid, Madrid, Spain

**Keywords:** *Populus*, epigenetics, growth-dormancy, methylation, phenology, chromatin remodeling

## Abstract

In temperate and boreal regions, perennial trees arrest cell division in their meristematic tissues during winter dormancy until environmental conditions become appropriate for their renewed growth. Release from the dormant state requires exposure to a period of chilling temperatures similar to the vernalization required for flowering in *Arabidopsis*. Over the past decade, genomic DNA (gDNA) methylation and transcriptome studies have revealed signatures of chromatin regulation during active growth and winter dormancy. To date, only a few chromatin modification genes, as candidate regulators of these developmental stages, have been functionally characterized in trees. In this work, we summarize the major findings of the chromatin-remodeling role during growth-dormancy cycles and we explore the transcriptional profiling of vegetative apical bud and stem tissues during dormancy. Finally, we discuss genetic strategies designed to improve the growth and quality of forest trees.

## Introduction

In temperate and boreal regions, a perennial plant’s interannual life cycle comprises multiple vegetative growth, and dormancy cycles. To guarantee survival, trees synchronize their growth and flowering times with the most favorable climate conditions of the year by following instructive information of annual photoperiod, and temperature patterns (Ding and Nilsson, 2016; Singh et al., 2016). For example, prior to winter, cell division in meristematic tissues is arrested and a protective structure is formed, i.e., the apical bud, in which a quiescent shoot apical meristem (SAM) and embryonic leaves are sheltered during the winter. In several tree species, such as poplar (*Populus* sp.), photoperiod plays a major role in cell division arrest, and bud formation (Fennell and Hoover, 1991; Cooke et al., 2012; Petterle et al., 2013). Such trees are able to sense the shortening of day length and thus anticipate the winter period. In other tree species, such as apple, growth cessation, and bud formation are controlled by temperature (Tanino et al., 2010). Once endodormancy has been established, low non-lethal temperatures progressively lead to dormancy release (chilling requirement). Once fulfilled, dormancy is released while growth cessation is maintained via external signals (ecodormancy), mainly low temperatures (Ding and Nilsson, 2016). Finally, spring growth-promoting temperatures produce bud break in vegetative buds, followed by vegetative growth.

These developmental processes require orchestration of specific temporal and spatial patterns of gene expression. Chromatin-modification-based regulation of gene expression during dormancy-growth cycles have been proposed to play a role in the organization of these patterns based on the identification of spatio-temporal patterns of epigenetic marks and the seasonal expression profiling of chromatin modification genes (Schrader et al., 2004; Ruttink et al., 2007; Santamaría et al., 2009; Karlberg et al., 2010; Conde et al., 2013, Conde et al., 2017a; Howe et al., 2015; Kumar et al., 2016). Epigenetic targets arise from covalent modifications of DNA and histones that will determine the accessibility of the transcription machinery to chromatin. Unlike in animals where epigenetics targets are established during embryonic development, in plants, epigenetic mechanisms also operate during post-embryonic developmental stages, contributing to plant developmental plasticity (Henderson and Jacobsen, 2007).

In this perspective article, we review the most recent evidences of DNA methylation and histone modification roles during annual growth-dormancy cycles in trees. In addition, we explore RNA-seq-based gene expression profiles in poplar vegetative apical bud and stem tissues, discovering seasonal expression patterns of genes involved in DNA methylation machinery. Finally, we discuss future strategies focused on chromatin remodeling for tree biotechnology applications.

## DNA Methylation and Growth-Dormancy Cycles

### DNA Methylation Patterns During Winter Dormancy

Genomic DNA methylation refers to the addition of a methyl group to the carbon atom at the fifth position of a cytosine (5 mC). DNA methylation plays a major role in gene expression, genome protection and stability through transposon silencing, DNA recombination, and other biological processes (Teixeira and Colot, 2010; Mirouze et al., 2012; Saze et al., 2012). Variation in DNA methylation has impact on plants phenotypic plasticity (Bossdorf et al., 2008; Bräutigam et al., 2013; Kooke et al., 2015). Several studies have revealed DNA methylation patterns during growth-dormancy cycles, both in buds, and stems. In chestnut, Santamaría et al. (2009) quantified higher levels of gDNA methylation and lower H4 acetylation levels in vegetative dormant apical buds compared to actively growing apices. Accordingly, poplar stems showed higher levels of gDNA methylation and lower levels of acetylation of lysine K8 of histone H4 during winter dormancy compared to active growth (Conde et al., 2013). Kumar et al. (2016) found that DNA methylation levels in apple decreased gradually from flower bud dormancy to fruit set. This dynamics in apple buds was only observed when apple trees were grown in environmental conditions satisfying the chilling requirement for winter dormancy release (Kumar et al., 2016). A search of differentially methylated genes in flower buds of almond by epi-Genotyping by sequencing (epi-GBS) led to a higher number of hypermethylated sequences in dormant buds when compared with dormancy-released samples (Prudencio et al., 2018). Recently, DNA methylation patterns during winter dormancy have been weekly examined in SAM tissue, from January to the time of vegetative bud break (Conde et al., 2017a). Results revealed a hypermethylation-hypomethylation wave formed by an initial stage of gDNA hypermethylation followed by a period of progressive 5 mC reduction to minimum levels before vegetative bud break and a 5 mC increase coincident with cell division reactivation (Conde et al., 2017a). Remarkably, a similar hypermethylation-hypomethylation wave has also been described in inflorescence SAM during the cold treatment of sugar beet (*Beta vulgaris*), suggesting comparable DNA methylation dynamics during vernalization and chilling requirements (Trap-Gentil et al., 2011; Conde et al., 2017a). Collectively, these findings suggest dynamic postembryonic deposition and removal of DNA methylation marks in SAM and stem tissues of woody perennials closely linked to the environmental factors.

### DNA Methylation Machinery Profile During Winter Dormancy

To investigate in poplar how the DNA methylation machinery could create this winter hypermethylation-hypomethylation wave in vegetative SAM and stem tissues, we performed a RNA-seq mediated gene expression profiling on weekly collected vegetative apical buds and stems of hybrid poplar (*Populus tremula* × *alba* INRA clone 717 1B4), grown under natural conditions in Pozuelo de Alarcón, Madrid, over the period of January 13th to April 14th 2015 for apical buds, and from November 7th 2014 to April 9th 2015 for stems, coinciding with bud break. Weekly time points were grouped according to the Pearson correlation for samples within the groups and considered as a biological replicate. A detailed list of sample names, dates and groups is shown in the Supplementary Table S1. These analyses resulted in 6 groups for apical bud samples, from mid-winter to mid-spring, and 10 groups for stem samples, from late-fall to mid-spring. The expression data of this experiment can be found in Phytozome^1^, under the expression tab for each gene.

**FIGURE 1:**
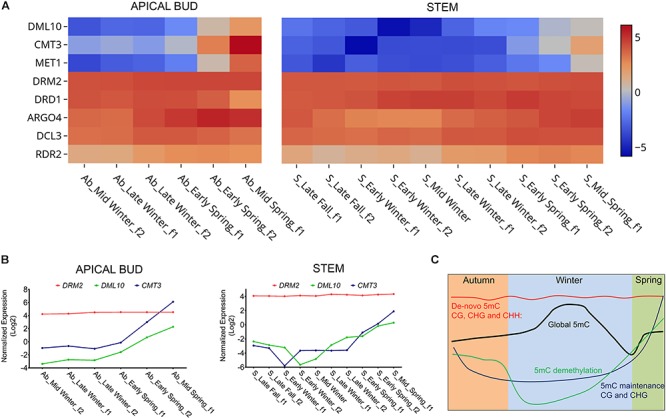
**(A)** Heat map showing normalized expression of genes coding for the enzymes involved in DNA methylation/demethylation during winter dormancy in hybrid poplar: *DML10* (Potri.010G234400), *CMT3* (Potri.001G009600), *MET1* (Potri.004G134000), *DRM2* (Potri.001G347000), *DRD1* (Potri.009G120700), *ARGO4* (Potri.001G219700), *DCL3* (Potri.018G047500), *RDR2* (Potri.015G073700), from mid-winter to mid-spring in apical bud, and from late-fall to mid-spring in stem. We added “early,” “mid,” or “late” to the name of groups when sampling dates were within the first, second, or third month of each season, following the Northern Meteorological Seasons dates. And “f1” or “f2,” for the fortnight-1 and fortnight-2 within the month. **(B)** Line plot showing normalized expression during winter dormancy of *CMT3*, *DRM2*, and *DML10* in apical vegetative bud and stem tissues. **(C)** Diagram showing the pattern of overall DNA methylation during hybrid poplar dormancy (taken from Conde et al., 2017a) along with patterns of the genes involved in DNA methylation/demethylation during this period.

Our RNAseq-based gene expression profiles revealed that the poplar homologs to *Arabidopsis* genes, involved in *de novo* DNA methylation machinery, such us *domains rearranged 2* (*DRM2*), are highly and constantly expressed from autumn to spring including winter dormancy in apical bud and stem tissues (Figure 1A,B). In contrast, a seasonal specific gene expression pattern was found for a plant specific 5-methyl-cytosine DEMETER-like demethylase (*DML10*). *DML10* showed a steady expression decline during early dormancy followed by a progressive increase in mRNA levels from mid-winter, with maximum expression observed at bud break in apical bud and stem tissues (Figure 1A,B). Similar results were reported in transcriptomics performed in poplar stem and lateral vegetative buds (Shim et al., 2014; Howe et al., 2015). In addition to that, poplar homologs to *methyltransferase 1* (*MET1*) and *chromomethylase 3* (*CMT3*), that operate in CG and CHG contexts, were found induced just before the onset of bud break in apical bud and stem tissues (Figure 1A,B). According to Shim et al. (2014), *CMT3* is also relatively highly expressed during growth resumption and active growth, while *MET1* is more expressed during endodormancy and the start of ecodormancy in stem tissues. This difference in *MET1* expression could be explained by the different environmental conditions in which the two experiments were carried out.

Collectively, these gene expression patterns evoke a plausible scenario whereby DNA methylation levels gradually increase during winter dormancy, likely via the unbalance between *de novo* DNA methylation and demethylation activity induced by the downregulation of *DML10* during early dormancy (Figure 1C). Since 5 mC shows a maximal accumulation during winter dormancy, the progressive decline in 5 mC observed at the end of the dormancy period correlates with the induction of *DML10* mRNA observed here (Figure 1C). In addition, the 5 mC increase produced during bud break was concurrent with the induction of *MET1* and *CMT3*. These enzymes could contribute to the maintenance of heterochromatin and transposon methylation during cell replication, once cell division has been reactivated in poplar SAM. Together, these findings highlight the main contribution of DML10 enzyme in generating winter specific 5 mC pattern.

In spite of the close agreement between DNA methylation levels and gene expression of methylation enzymes, we cannot discard putative developmental, and tissue-dependent effects on the transcriptional and post-transcriptional regulation of *DML10* and other epigenetic modifier genes, with potential impact on the methylation status of particular tissues and cells. In order to clarify this, tissue and cell-specific analyses would be very helpful.

### DNA Methylation and Energy Status During Winter Dormancy

DNA methyltransferases use S-adenosyl methionine (SAMe) as a methyl group donor (Lyko, 2018). The end-product of this catalytic reaction is S-adenosyl homocysteine (SAH). SAH hydrolase (SAHH) breaks down SAH into adenosine and homocysteine, the precursor of methionine (Met), which in turn produces SAMe. SAHH activity is linked to cell metabolism, as SAHH activity is dependent of NAD^+^ (Grillo and Colombatto, 2008). During glycolysis and TCA cycles, in which cells extract energy from glucose and pyruvate breakdown, molecules of NAD^+^ are reduced into NADH, and hence a lower NAD^+^/NADH ratio could diminish the activity of SAHH (Luo and Kuo, 2009). In apple, the activity of the TCA cycle enzyme isocitrate dehydrogenase (ICDH) was found to be low in dormant vegetative buds compared to non-dormant buds (Wang et al., 1991). Transcriptional profiling of *Paoenia ostii* highlighted the importance of glycolysis and TCA cycle induction for flower bud dormancy release (Gai et al., 2013). We suggest that SAHH activity could acts as the bridge between the energetic status and DNA methylation, contributing to the DNA methylation increase observed during winter dormancy, following the NAD^+^/NADH ratio increment. We identified two poplar genes coding for SAHH enzymes: SAHH1 (Potri.001G320500) and SAHH17 (Potri.017G059400). Interestingly, both genes have been genetically associated with vegetative bud phenology during dormancy by Evans et al. (2014).

## Histone Modification and Growth-Dormancy Cycles

### Gene-Specific Histone Marks in Buds

The modification of histones via different biochemical mechanisms and chromatin remodeling are key elements of plant development regulation and their response to environmental conditions (de la Paz Sanchez et al., 2015). In particular, histone modifications identified in *dormancy associated MADS-box* (*DAM*) genes, the master regulators of vegetative and reproductive bud dormancy in Rosaceae and other perennial species, resemble chromatin dynamics of known regulators of vernalization, and seed dormancy and germination in *Arabidopsis* (Rios et al., 2014; Velappan et al., 2017). *DAM1-6* was first identified in peach as a family of six tandemly arrayed genes coding for similar MADS-Box transcription factors, which were partially deleted in the *evergrowing* non-dormant mutant of peach (Bielenberg et al., 2008). Related *DAM* genes involved in bud dormancy maintenance have been also described in leafy spurge (Horvath et al., 2010), Japanese apricot (Sasaki et al., 2011), pear (Niu et al., 2016), and apple (Wu et al., 2017) among other species. *DAM* genes have been postulated to modulate dormancy via transcriptional regulation of *FLOWERING LOCUS T* (Hsu et al., 2011) in vegetative buds of leafy spurge (Hao et al., 2015) and flower buds of pear (Niu et al., 2016), and also the biosynthesis of ABA in lateral flower buds of pear (Tuan et al., 2017). Concomitant with bud dormancy release, a decrease in the trimethylation of lysine 4 in histone H3 (H3K4me3) has been found in the chromatin of the *DAM1* gene in leafy spurge (Horvath et al., 2010), *DAM6* in peach (Leida et al., 2012), and *PpMADS13-1* in pear (Saito et al., 2015), suggesting that vegetative and flower buds share chromatin-related modifications across dormancy development in different species. Moreover, in peach, we observed a decrease in H3 acetylation on the ATG region of *DAM6*, and an increase in trimethylation of lysine 27 in H3 (H3K27me3) in a wider region of the gene (Leida et al., 2012). These chromatin changes are commonly associated with gene repression, and in fact, coincide with down-regulation of *DAM*-like genes, suggesting a mechanism for flower bud dormancy modulation through an arranged succession of epigenetic events in chromatin of *DAM* genes (Rios et al., 2014). In addition, small interference RNAs and microRNAs have been also postulated to regulate *DAM*-like expression and the floral dormancy transition in sweet cherry and pear, respectively (Niu et al., 2016; Rothkegel et al., 2017).

**FIGURE 2:**
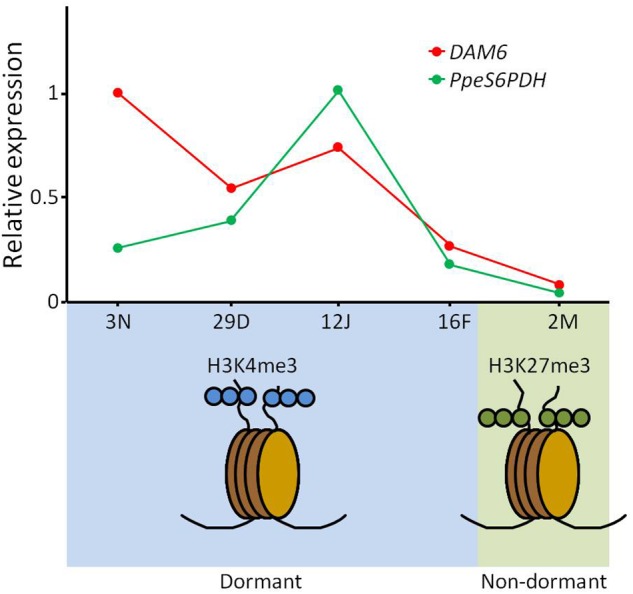
Chromatin modifications produced in *DAM6* and *PpeS6PDH* genes during bud development in peach. Relative expression levels of *DAM6* and *PpeS6PDH* in flower buds are shown in the upper panel. Enriched histone modifications in dormant (blue area) and dormancy-released (green area) buds in *DAM6* and *PpeS6PDH* are depicted in the lower panel.

However, *DAM* genes are not the only known targets of chromatin modification during bud dormancy and development. The *early bud-break 1* (*EBB1*) gene encodes a putative APETALA2/Ethylene responsive transcription factor that reactivates vegetative meristem growth and bud-break after dormancy release in poplar (Yordanov et al., 2014), with conserved orthologs in other woody perennial species (Busov et al., 2016). Two regions on the promoter and ATG site of *PpEBB* gene from pear have been found differentially trimethylated at H3K4 in accordance with *PpEBB* up-regulation during the floral sprouting stage (Tuan et al., 2016). On the other hand, *PpeS6PDH* encodes a sorbitol-6-phosphate dehydrogenase involved in the synthesis of sorbitol in axillary flower dormant buds of peach (Lloret et al., 2017). *PpeS6PDH* expression was found to be silenced in dormancy-released buds concomitantly with an H3K4me3 decrease and H3K27me3 increase in a particular regulatory region of the gene near the translation start (Figure 2). We postulated that sorbitol exerts a role as cryoprotectant and compatible solute in dormant buds, and hence dormancy regulation by *DAM6* and abiotic stress tolerance by *PpeS6PDH* could share a common mechanism for gene repression through concerted H3K27 trimethylation (Lloret et al., 2018).

### Histone Modifiers During Winter Dormancy

Trimethylation of H3K27 is achieved by the polycomb repressive complex 2 (PRC2), containing components conserved in animals and plants (Alvarez-Venegas, 2010; Derkacheva and Hennig, 2014). In peach, several genes coding for such subunits of PRC2 complexes co-localize with quantitative trait loci for the chilling requirement and bloom date traits, providing genetic evidence of the role of these complexes in dormancy regulation (Zhebentyayeva et al., 2014). The PRC2 component gene *fertilization independent endosperm* (*FIE*) was sharply up-regulated under short-photoperiod treatments correlating with growth cessation and dormancy induction in poplar (Ruttink et al., 2007). Moreover, *FIE* suppression by RNAi prevented the establishment of dormancy in transgenic hybrid aspen (*Populus* × spp.), even though growth cessation and bud formation were not affected (Petterle, 2011). In peach buds, genome-wide stretches enriched in H3K27me3 were found associated with GA-repeat sequences (de la Fuente et al., 2015), suggesting that basic pentacysteine (BPC) factors able to bind GA-repeats could mediate the recruitment of PRC2 and thus H3K27me3 modification in flower bud dormancy dependent genes such as *DAM* family and *PpeS6PDH*, as was recently reported in *Arabidopsis* (Xiao et al., 2017).

Additional chromatin-related genes, such as the chromatin remodeler *PICKLE* (*PKL*), and putative modifiers involved in histone deacetylation (*HDA14* and *HDA08*), histone lysine methylation (*SUVR3*), and histone ubiquitination (*HUB2*) are also up-regulated during the transition to dormancy in *Populus* (Ruttink et al., 2007; Karlberg et al., 2010). Interestingly, down-regulation of *PKL* expression restores photoperiod-induced dormancy in *abi1-1* hybrid aspen mutants with a defective abscisic acid (ABA) response, suggesting that ABA promotes dormancy by repressing *PKL* (Tylewicz et al., 2018).

In axillary flower buds, dormancy changes are concomitant with flower developmental processes such as gametogenesis and organ development, which thus may account for part of the observed regulation of modifier genes and histone modifications. Strong changes in gene expression associated with microsporogenesis have been found in peach in parallel to dormancy release (Ríos et al., 2013), although no histone changes have been reported so far during this or other developmental processes in trees. However, in the model species *Arabidopsis* and rice, several epigenetic mechanisms involving chromatin remodeling and histone modification by PRC2 and other complexes modulate floral initiation and development at different steps (Guo et al., 2015), suggesting that processes other than dormancy may contribute to modify gene expression, DNA methylation, and histone modifications measurements in tree buds. Detailed tissue and organ-specific studies will be required in order to assess the contribution of particular organs and processes to these biochemical and molecular observations.

## Biotechnology of the Epigenome in Trees

Modification of the plant epigenome contributes substantially to variation in plant growth, morphology, and plasticity (Johannes et al., 2008). In temperate and boreal trees, several lines of evidence point to environmental-guided DNA and histone modification profiles as critical regulators of chromatin function controlling the tempo of annual growth-dormancy cycles.

Recently, Kumar et al. (2016) reported that DNA demethylation in apple trees, which precedes floral bud break and fruit formation, only occurs under environments that fulfill the chilling requirement. Moreover, Conde et al. (2017a) observed that the induction of *DML10* expression before poplar bud break only happens if a chilling requirement has been fulfilled. These observations indicate that the downregulation of 5 mC observed during winter dormancy is closely linked to the transition from endodormacy to ecodormancy and is a precondition for growth resumption of vegetative and reproductive meristems. Therefore, modification of this DNA methylation pattern may have an impact on phenology as a biotechnological strategy to relax or tighten the chromatin state thus modifying the annual growth-dormancy cycle in trees.

Based on the hyper-hypomethylation wave of 5 mC reported during winter in poplar, it could be hypothesized that disruption of this pattern by creating hypermethylated poplar lines will delay growth resumption after dormancy. Conversely the generation of hypomethylated lines could result in rapid growth resumption following dormancy period. Candidate genes that alter winter dormancy DNA methylation/demethylation patterns can be inferred from our analyses. Thus, it could be possible to engineer poplars showing DNA hypermethylation through the upregulation of *DRM2* or silencing of *DML10* or *SAHH*, respectively. On the other hand, upregulation of DML10 or SAHH, or silencing of DRM2, respectively, could yield hypomethylated poplar lines. Accordingly, Conde et al. (2017a) described an RNAi strategy to reduce *DML* gene expression in hybrid poplars, noting that transgenic poplars featuring *DML10* downregulation showed significantly higher levels of DNA methylation, which delayed bud break. These RNAi *DML10* poplar lines showed negligible alteration of growth and development despite the specific effect mentioned. Equally, the overexpression of a chestnut *DML* (*Castanea sativa*) in hybrid poplar resulted in transgenic lines in which apical bud formation during dormancy establishment was accelerated showing no other visible alterations (Conde et al., 2017b). Hence, the consequences of epigenome engineering need to be tested gene by gene to check for possible pleiotropic phenotypes. Alternatively, a detailed knowledge of the tissue and time-dependent expression and activity of key modifier genes may provide useful information for the specific activation/repression of epigenetic regulators without undesirable effects on many other biological processes affected by them, with the help of adequate specific promoters.

The functional role of histone modifications during tree annual growth-dormancy cycles needs further clarification. The PRC2 complex seems to play a key role in dormancy regulation through the H3K27me3 modification on regulatory genes, although only the function of the PRC2 component FIE has been initially explored in poplar (Petterle, 2011). Tree orthologs of other subunits of this complex, such as *CURLY LEAF* (*CLF*) and *SWINGER* (*SWN*) could provide additional evidences on PCR2 participation in dormancy mechanisms and also serve as candidates for phenological manipulation. However, the putative targets of PCR2 activity may constitute more suitable and specific objectives for biotechnological approaches.

A genomic deletion of several *DAM* genes located in tandem causes a non-dormant phenotype in peach, highlighting the functional relevance of these genes in regulating dormancy (Bielenberg et al., 2008). As *DAM* genes have been postulated to integrate environmental signals, particularly chilling accumulation, by an epigenetic mechanism involving H3K27me3 and other histone modifications, targeted mutants on *DAM cis*-elements promoting the binding of histone modifier complexes could be employed to specifically modulate the response of *DAM* genes to chilling, with a potential use in manipulating the adaptability of stone fruit crops to changing climatic conditions. GA repeats on the large intron of *DAM6* may serve as tentative candidate *cis*-elements for this approach. In addition to GA repeats, the telobox *cis*-element has been shown to recruit the PRC2 complex in *Arabidopsis* through telomere-repeat-binding factors (TRBs) (Zhou et al., 2018). Thus, putative telobox motifs on *DAM* and other regulatory genes could also be modified in order to reprogram the environmental input on dormancy cycles.

Additional research is needed on the study of detailed characterization of the seasonal growth cycles involving frequently sampled intervals along the growth-dormancy-growth cycle. Detailed, subtle changes in chromatin modifications may be occurring outside the published data sets. In addition, new analytical techniques are emerging that may allow high-throughput characterization of DNA methylation and histone acetylation (e.g., ATAC-seq) should be applied to such periodically sampled tissues.

Other genes mentioned in this study involved in DNA methylation, RNA interference, chromatin remodeling, histone modification and transcriptional regulation of meristem growth, and dormancy are interesting candidates for biotechnological applications in tree phenology. The CRISPR/Cas9 system emerges as a promising technique due to its simplicity, design flexibility and high degree of efficiency. However, so far, few studies involving the use of this technique in tree species have been conducted, and no study has examined the impacts of epigenetics mark modifications (Fan et al., 2015; Zhou et al., 2015). Future studies designed to edit specific epigenetic regulator genes will unravel the impacts of particular epigenetic modifications on the annual cycle of trees.

## Author Contributions

DC, MP, AL, MB, PG-M, GR, and IA participated in the discussions described here. DC, MP, AS, GT, and IA implicated in the RNA-seq analysis. DC, MP, GT, GR, and IA wrote the manuscript.

## Conflict of Interest Statement

The authors declare that the research was conducted in the absence of any commercial or financial relationships that could be construed as a potential conflict of interest.

## Footnotes

1 https://phytozome.jgi.doe.gov/pz/portal.html

## References

[B1] Alvarez-Venegas R. (2010). Regulation by polycomb and trithorax group proteins in *Arabidopsis*. *Arabidopsis Book* 8:e0128. 10.1199/tab.0128 22303254 PMC3244960

[B2] Bielenberg D. G. Wang Y. Li Z. Zhebentyayeva T. Fan S. Reighard G. L. (2008). Sequencing and annotation of the evergrowing locus in peach [*Prunus persica (L.) Batsch] reveals a cluster of six MADS-box transcription factors as candidate genes for regulation of terminal bud formation*. *Tree Genet. Genomes* 4 495–507. 10.1007/s11295-007-0126-9

[B3] Bossdorf O. Richards C. L. Pigliucci M. (2008). Epigenetics for ecologists. *Ecol. Lett.* 11 106–115. 10.1111/j.1461-0248.2007.01130.x 18021243

[B4] Bräutigam K. Vining K. J. Lafon-Placette C. Fossdal C. G. Mirouze M. Marcos J. G. (2013). Epigenetic regulation of adaptive responses of forest tree species to the environment. *Ecol. Evol.* 3 399–415. 10.1002/ece3.461 23467802 PMC3586649

[B5] Busov V. Carneros E. Yakovlev I. (2016). EARLY BUD-BREAK1 (EBB1) defines a conserved mechanism for control of bud-break in woody perennials. *Plant Signal. Behav.* 11:e1073873. 10.1080/15592324.2015.1073873 26317150 PMC4883858

[B6] Conde D. González-Melendi P. Allona I. (2013). Poplar stems show opposite epigenetic patterns during winter dormancy and vegetative growth. *Trees Struct. Funct.* 27 311–320. 10.1007/s00468-012-0800-x

[B7] Conde D. Le Gac A.-L. Perales M. Dervinis C. Kirst M. Maury S. (2017a). Chilling responsive DEMETER-LIKE DNA demethylase mediates in poplar bud break. *Plant Cell Environ.* 40 2236–2249. 10.1111/pce.13019 28707409

[B8] Conde D. Moreno A. Christopher C. José D. Sánchez M. R. Kirst M. (2017b). Overexpression of DEMETER, a DNA demethylase, promotes early apical bud maturation in poplar. *Plant Cell Environ.* 40 2806–2819. 10.1111/pce.13056 28810288

[B9] Cooke J. E. K. Eriksson M. E. Junttila O. (2012). The dynamic nature of bud dormancy in trees: environmental control and molecular mechanisms. *Plant Cell Environ.* 35 1707–1728. 10.1111/j.1365-3040.2012.02552.x 22670814

[B10] de la Fuente L. Conesa A. Lloret A. Badenes M. L. Ríos G. (2015). Genome-wide changes in histone H3 lysine 27 trimethylation associated with bud dormancy release in peach. *Tree Genet. Genomes* 11:45 10.1007/s11295-015-0869-7

[B11] de la Paz Sanchez M. Aceves-García P. Petrone E. Steckenborn S. Vega-León R. Álvarez-Buylla E. R. (2015). The impact of Polycomb group (PcG) and Trithorax group (TrxG) epigenetic factors in plant plasticity. *New Phytol.* 208 684–694. 10.1111/nph.13486 26037337

[B12] Derkacheva M. Hennig L. (2014). Variations on a theme: polycomb group proteins in plants. *J. Exp. Bot.* 65 2769–2784. 10.1093/jxb/ert410 24336446

[B13] Ding J. Nilsson O. (2016). Molecular regulation of phenology in trees-because the seasons they are a-changin’. *Curr. Opin. Plant Biol.* 29 73–79. 10.1016/j.pbi.2015.11.007 26748352

[B14] Evans L. M. Slavov G. T. Rodgers-Melnick E. Martin J. Ranjan P. Muchero W. (2014). Population genomics of *Populus trichocarpa identifies signatures of selection and adaptive trait associations*. *Nat. Genet.* 46 1089–1096. 10.1038/ng.3075 25151358

[B15] Fan D. Liu T. Li C. Jiao B. Li S. Hou Y. (2015). Efficient CRISPR/Cas9-mediated targeted mutagenesis in Populus in the first generation. *Sci. Rep.* 5:12217. 10.1038/srep12217 26193631 PMC4507398

[B16] Fennell A. Hoover E. (1991). Photoperiod influences growth bud dormancy and cold acclimation in *Vitis labruscana* and *Vitis riparia.* *J. Am. Soc. Hortic. Sci.* 116 270–273. 10.21273/jashs.116.2.270

[B17] Gai S. Zhang Y. Liu C. Zhang Y. Zheng G. (2013). Transcript profiling of *Paeonia ostii during artificial chilling induced dormancy release identifies activation of GA pathway and carbohydrate metabolism*. *PLoS One* 8:e55297. 10.1371/journal.pone.0055297 23405132 PMC3566188

[B18] Grillo M. A. Colombatto S. (2008). S-adenosylmethionine and its products. *Amino Acids* 34 187–193. 10.1007/s00726-007-0500-9 17334902

[B19] Guo S. Sun B. Looi L. S. Xu Y. Gan E. S. Huang J. (2015). Co-ordination of flower development through epigenetic regulation in two model species: rice and *Arabidopsis*. *Plant Cell Physiol.* 56 830–842. 10.1093/pcp/pcv037 25746984

[B20] Hao X. Chao W. Yang Y. Horvath D. (2015). Coordinated expression of FLOWERING LOCUS T and DORMANCY ASSOCIATED MADS-BOX-like genes in leafy spurge. *PLoS One* 10:e0126030. 10.1371/journal.pone.0126030 25961298 PMC4427404

[B21] Henderson I. R. Jacobsen S. E. (2007). Epigenetic inheritance in plants. *Nature* 447 418–424. 10.1038/nature05917 17522675

[B22] Horvath D. P. Sung S. Kim D. Chao W. Anderson J. (2010). Characterization, expression and function of DORMANCY ASSOCIATED MADS-BOX genes from leafy spurge. *Plant Mol. Biol.* 73 169–179. 10.1007/s11103-009-9596-5 20066557

[B23] Howe G. T. Horvath D. P. Dharmawardhana P. Priest H. D. Mockler T. C. Strauss S. H. (2015). Extensive transcriptome changes during natural onset and release of vegetative bud dormancy in Populus. *Front. Plant Sci.* 6:989. 10.3389/fpls.2015.00989 26734012 PMC4681841

[B24] Hsu C. Y. Adams J. P. Kim H. No K. Ma C. Strauss S. H. (2011). FLOWERING LOCUS T duplication coordinates reproductive and vegetative growth in perennial poplar. *Proc. Natl. Aacd. Sci. U.S.A.* 108 10756–10761. 10.1073/pnas.1104713108 21653885 PMC3127867

[B25] Johannes F. Colot V. Jansen R. C. (2008). Epigenome dynamics: a quantitative genetics perspective. *Nat. Rev. Genet.* 9 883–890. 10.1038/nrg2467 18927581

[B26] Karlberg A. Englund M. Petterle A. Molnar G. Sjödin A. Bako L. (2010). Analysis of global changes in gene expression during activity-dormancy cycle in hybrid aspen apex. *Plant Biotechnol.* 27 1–16. 10.5511/plantbiotechnology.27.1

[B27] Kooke R. Johannes F. Wardenaar R. Becker F. Etcheverry M. Colot V. (2015). Epigenetic basis of morphological variation and phenotypic plasticity in *Arabidopsis thaliana*. *Plant Cell* 27 337–348. 10.1105/tpc.114.133025 25670769 PMC4456930

[B28] Kumar G. Rattan U. K. Singh A. K. (2016). Chilling-mediated DNA methylation changes during dormancy and its release reveal the importance of epigenetic regulation during winter dormancy in apple (*Malus x *domestica* Borkh.)*. *PLoS One* 11:e0149934. 10.1371/journal.pone.0149934 26901339 PMC4763039

[B29] Leida C. Conesa A. Llácer G. Badenes M. L. Ríos G. (2012). Histone modifications and expression of DAM6 gene in peach are modulated during bud dormancy release in a cultivar-dependent manner. *New Phytol.* 193 67–80. 10.1111/j.1469-8137.2011.03863.x 21899556

[B30] Lloret A. Badenes M. L. Ríos G. (2018). Modulation of dormancy and growth responses in reproductive buds of temperate trees. *Front. Plant Sci.* 9:1368. 10.3389/fpls.2018.01368 30271422 PMC6146825

[B31] Lloret A. Martínez-Fuentes A. Agustí M. Badenes M. L. Ríos G. (2017). Chromatin-associated regulation of sorbitol synthesis in flower buds of peach. *Plant Mol. Biol.* 95 507–517. 10.1007/s11103-017-0669-6 29038917

[B32] Luo J. Kuo M.-H. (2009). Linking nutrient metabolism to epigenetics. *Cell Sci. Rev.* 6. 1.

[B33] Lyko F. (2018). The DNA methyltransferase family: a versatile toolkit for epigenetic regulation. *Nat. Rev. Genet.* 19 81–92. 10.1038/nrg.2017.80 29033456

[B34] Mirouze M. Lieberman-Lazarovich M. Aversano R. Bucher E. Nicolet J. Reinders J. (2012). Loss of DNA methylation affects the recombination landscape in *Arabidopsis*. *Proc. Natl. Acad. Sci. U.S.A.* 109 5880–5885. 10.1073/pnas.1120841109 22451936 PMC3326504

[B35] Niu Q. Li J. Cai D. Qian M. Jia H. Bai S. (2016). Dormancy-associated MADS-box genes and microRNAs jointly control dormancy transition in pear (*Pyrus pyrifolia white pear group) flower bud*. *J. Exp. Bot.* 67 239–257. 10.1093/jxb/erv454 26466664 PMC4682432

[B36] Petterle A. (2011). *ABA and Chromatin Remodeling Regulate the Activity-Dormancy Cycle in Hybrid Aspen.* Ph.D. thesis, Swedish University of Agricultural Sciences, Uppsala.

[B37] Petterle A. Karlberg A. Bhalerao R. P. (2013). Daylength mediated control of seasonal growth patterns in perennial trees. *Curr. Opin. Plant Biol.* 16 301–306. 10.1016/j.pbi.2013.02.006 23473967

[B38] Prudencio Á. S. Werner O. Martínez-García P. J. Dicenta F. Ros R. M. Martínez-Gómez P. (2018). DNA methylation analysis of dormancy release in almond (*Prunus dulcis) flower buds using epi-genotyping by sequencing*. *Int. J. Mol. Sci.* 19:E3542. 10.3390/ijms19113542 30423798 PMC6274898

[B39] Rios G. Leida C. Conejero A. Badenes M. L. (2014). Epigenetic regulation of bud dormancy events in perennial plants. *Front. Plant Sci.* 5:247. 10.3389/fpls.2014.00247 24917873 PMC4042555

[B40] Ríos G. Tadeo F. R. Leida C. Badenes M. L. (2013). Prediction of components of the sporopollenin synthesis pathway in peach by genomic and expression analyses. *BMC Genomics* 14:40. 10.1186/1471-2164-14-40 23331975 PMC3556096

[B41] Rothkegel K. Sánchez E. Montes C. Greve M. Tapia S. Bravo S. (2017). DNA methylation and small interference RNAs participate in the regulation of MADS-box genes involved in dormancy in sweet cherry (*Prunus avium L.)*. *Tree Physiol.* 37 1739–1751. 10.1093/treephys/tpx055 28541567

[B42] Ruttink T. Arend M. Morreel K. Storme V. Rombauts S. Fromm J. (2007). A molecular timetable for apical bud formation and dormancy induction in poplar. *Plant Cell* 19 2370–2390. 10.1105/tpc.107.052811 17693531 PMC2002631

[B43] Saito T. Bai S. Imai T. Ito A. Nakajima I. Moriguchi T. (2015). Histone modification and signalling cascade of the dormancy-associated MADS-box gene, PpMADS13-1, in Japanese pear (*Pyrus pyrifolia) during endodormancy*. *Plant Cell Environ.* 38 1157–1166. 10.1111/pce.12469 25311427

[B44] Santamaría M. Hasbún R. Valera M. Meijón M. Valledor L. Rodríguez J. L. (2009). Acetylated H4 histone and genomic DNA methylation patterns during bud set and bud burst in *Castanea sativa*. *J. Plant Physiol.* 166 1360–1369. 10.1016/j.jplph.2009.02.014 19376609

[B45] Sasaki R. Yamane H. Ooka T. Jotatsu H. Kitamura Y. Akagi T. (2011). Functional and expressional analyses of PmDAM genes associated with endodormancy in Japanese apricot. *Plant Physiol.* 157 485–497. 10.1104/pp.111.181982 21795580 PMC3165894

[B46] Saze H. Tsugane K. Kanno T. Nishimura T. (2012). DNA methylation in plants: relationship to small RNAs and histone modifications, and functions in transposon inactivation. *Plant Cell Physiol.* 53 766–784. 10.1093/pcp/pcs008 22302712

[B47] Schrader J. Moyle R. Bhalerao R. Hertzberg M. Lundeberg J. Nilsson P. (2004). Cambial meristem dormancy in trees involves extensive remodelling of the transcriptome. *Plant J.* 40 173–187. 10.1111/j.1365-313X.2004.02199.x 15447645

[B48] Shim D. Ko J.-H. Kim W.-C. Wang Q. Keathley D. E. Han K.-H. (2014). A molecular framework for seasonal growth-dormancy regulation in perennial plants. *Hortic. Res.* 1:14059. 10.1038/hortres.2014.59 26504555 PMC4591672

[B49] Singh R. K. Svystun T. Aldahmash B. Jonsson A. M. Bhalerao R. P. (2016). Photoperiod- and temperature-mediated control of phenology in trees – a molecular perspective. *New Phytol.* 213 511–524. 10.1111/nph.14346 27901272

[B50] Tanino K. K. Kalcsits L. Silim S. Kendall E. Gray G. R. (2010). Temperature-driven plasticity in growth cessation and dormancy development in deciduous woody plants: a working hypothesis suggesting how molecular and cellular function is affected by temperature during dormancy induction. *Plant Mol. Biol.* 73 49–65. 10.1007/s11103-010-9610-y 20191309

[B51] Teixeira F. K. Colot V. (2010). Repeat elements and the *Arabidopsis DNA methylation landscape*. *Heredity* 105 14–23. 10.1038/hdy.2010.52 20461104

[B52] Trap-Gentil M. V. Hébrard C. Lafon-Placette C. Delaunay A. Hagège D. Joseph C. (2011). Time course and amplitude of DNA methylation in the shoot apical meristem are critical points for bolting induction in sugar beet and bolting tolerance between genotypes. *J. Exp. Bot.* 62 2585–2597. 10.1093/jxb/erq433 21227931

[B53] Tuan P. A. Bai S. Saito T. Imai T. Ito A. Moriguchi T. (2016). Involvement of EARLY BUD-BREAK, an AP2/ERF transcription factor gene, in bud break in Japanese pear (*Pyrus pyrifolia Nakai) lateral flower buds: expression, histone modifications and possible target genes*. *Plant Cell Physiol.* 57 1038–1047. 10.1093/pcp/pcw041 26940832

[B54] Tuan P. A. Bai S. Saito T. Ito A. Moriguchi T. (2017). *Dormancy-associated MADS-Box (DAM*) and the abscisic acid pathway regulate pear endodormancy through a feedback mechanism. *Plant Cell Physiol.* 58 1378–1390. 10.1093/pcp/pcx074 28586469

[B55] Tylewicz S. Petterle A. Marttila S. Miskolczi P. Azeez A. Singh R. K. (2018). Photoperiodic control of seasonal growth is mediated by ABA acting on cell-cell communication. *Science* 360 212–215. 10.1126/science.aan8576 29519919

[B56] Velappan Y. Signorelli S. Considine M. J. (2017). Cell cycle arrest in plants: what distinguishes quiescence, dormancy and differentiated G1? *Ann. Bot.* 120 495–509. 10.1093/aob/mcx082 28981580 PMC5737280

[B57] Wang S. Y. Jiao H. J. Faust M. (1991). Changes in metabolic enzyme activities during thidiazuron-induced lateral budbreak of apple. *Hortscience* 26 171–173. 10.21273/hortsci.26.2.171

[B58] Wu R. Tomes S. Karunairetnam S. Tustin S. D. Hellens R. P. Allan A. C. (2017). SVP-like MADS box genes control dormancy and budbreak in apple. *Front. Plant Sci.* 8:477. 10.3389/fpls.2017.00477 28421103 PMC5378812

[B59] Xiao J. Jin R. Yu X. Shen M. Wagner J. D. Pai A. (2017). *Cis and trans* determinants of epigenetic silencing by Polycomb repressive complex 2 in *Arabidopsis.* *Nat. Genet.* 49 1546–1552. 10.1038/ng.3937 28825728

[B60] Yordanov Y. S. Ma C. Strauss S. H. Busov V. B. (2014). EARLY BUD-BREAK 1 (EBB1) is a regulator of release from seasonal dormancy in poplar trees. *Proc. Natl. Acad. Sci. U.S.A.* 111 10001–10006. 10.1073/pnas.1405621111 24951507 PMC4103365

[B61] Zhebentyayeva T. N. Fan S. Chandra A. Bielenberg D. G. Reighard G. L. Okie W. R. (2014). Dissection of chilling requirement and bloom date QTLs in peach using a whole genome sequencing of sibling trees from an F2 mapping population. *Tree Genet. Genomes* 10 35–51. 10.1007/s11295-013-0660-6

[B62] Zhou X. Jacobs T. B. Xue L. J. Harding S. A. Tsai C. J. (2015). Exploiting SNPs for biallelic CRISPR mutations in the outcrossing woody perennial *Populus reveals 4-coumarate: CoA ligase specificity and redundancy*. *New Phytol.* 208 298–301. 10.1111/nph.13470 25970829

[B63] Zhou Y. Wang Y. Krause K. Yang T. Dongus J. A. Zhang Y. (2018). Telobox motifs recruit CLF/SWN-PRC2 for H3K27me3 deposition via TRB factors in *Arabidopsis*. *Nat. Genet.* 50 638–644. 10.1038/s41588-018-0109-9 29700471

